# β_2_ adrenergic agonist suppresses eosinophil-induced epithelial-to-mesenchymal transition of bronchial epithelial cells

**DOI:** 10.1186/s12931-017-0563-4

**Published:** 2017-05-02

**Authors:** Keigo Kainuma, Tetsu Kobayashi, Corina N. D’Alessandro-Gabazza, Masaaki Toda, Taro Yasuma, Kota Nishihama, Hajime Fujimoto, Yu Kuwabara, Koa Hosoki, Mizuho Nagao, Takao Fujisawa, Esteban C. Gabazza

**Affiliations:** 1 Allergy Center, Mie National Hospital, 357 Osato-kubota, Tsu, Mie 514-0125 Japan; 2 Department of Immunology, Mie University Graduate School of Medicine, Edobashi 2-174, Tsu, Mie 514-8507 Japan; 3 Department of Pulmonary and Critical Care Medicine, Mie University Graduate School of Medicine, Edobashi 2-174, Tsu, Mie 514-8507 Japan

**Keywords:** Asthma, Eosinophils, Epithelial cells, Integrins

## Abstract

**Background:**

Epithelial-mesenchymal transition is currently recognized as an important mechanism for the increased number of myofibroblasts in cancer and fibrotic diseases. We have already reported that epithelial-mesenchymal transition is involved in airway remodeling induced by eosinophils. Procaterol is a selective and full β_2_ adrenergic agonist that is used as a rescue of asthmatic attack inhaler form and orally as a controller. In this study, we evaluated whether procaterol can suppress epithelial-mesenchymal transition of airway epithelial cells induced by eosinophils.

**Methods:**

Epithelial-mesenchymal transition was assessed using a co-culture system of human bronchial epithelial cells and primary human eosinophils or an eosinophilic leukemia cell line.

**Results:**

Procaterol significantly inhibited co-culture associated morphological changes of bronchial epithelial cells, decreased the expression of vimentin, and increased the expression of E-cadherin compared to control. Butoxamine, a specific β_2_-adrenergic antagonist, significantly blocked changes induced by procaterol. In addition, procaterol inhibited the expression of adhesion molecules induced during the interaction between eosinophils and bronchial epithelial cells, suggesting the involvement of adhesion molecules in the process of epithelial-mesenchymal transition. Forskolin, a cyclic adenosine monophosphate-promoting agent, exhibits similar inhibitory activity of procaterol.

**Conclusions:**

Overall, these observations support the beneficial effect of procaterol on airway remodeling frequently associated with chronic obstructive pulmonary diseases.

## Background

Obstructive pulmonary diseases such as bronchial asthma and chronic obstructive pulmonary disease are chronic inflammation of the airways that are frequently associated with lung structural changes, termed airway remodeling [1, 2]. The pathogenesis of airway remodeling has not been fully elucidated. It may be a consequence of airway inflammation [3, 4]. β_2_ adrenergic agonists are not only the first line drug for relief of acute asthma symptoms but a long-term controller in combination with inhaled corticosteroids. Procaterol is a selective and full β_2_ adrenergic agonist that is used as a rescue of asthmatic attack in inhaler form and orally as a controller [5]. Studies in vitro have shown that β_2_ selective-agonists exert anti-inflammatory activity. β_2_ selective-agonists increase cyclic AMP levels, which inhibit mast cell and eosinophil degranulation, apoptosis and cytokine production [6–9]. Procaterol can also reduce the expression of adhesion molecules [6, 10]. A previous study has shown that systemic administration of tulobuterol, a β_2_-selective agonist, decreases eosinophil adhesion to endothelial cells resulting in reduction of eosinophil inflammation [11]. β_2_ adrenergic agonists are also very effective bronchodilators in COPD and they are part of the therapeutic strategy for the management of COPD patients [12, 13]. Short acting or long acting β_2_ agonists are administered in clinical practice through inhaler devices whose delivery efficiency has substantially improved by the use of computational models [13–17].

Epithelial to mesenchymal transition (EMT) leads to increased number of myofibroblasts in cancer and fibrotic diseases [18]. Eosinophils can cause airway remodeling by promoting EMT [19]. Recently, we and others have reported that direct contact of eosinophils with bronchial epithelial cells increases the expression of TGF-β_1_ leading to induction of EMT [20]. In the present study, we hypothesized that procaterol can suppress EMT of airway epithelial cells induced by eosinophils.

## Methods

### Reagent

L-glutamine, penicillin/streptomycin, donkey anti-mouse IgG-Alexa Fluor 488, Chicken anti-rabbit IgG-Alexa Fluor 594, Laemmli sample buffer and Trizol Reagent were purchased from Invitrogen (Carlsbad, CA). Dulbecco’s modified Eagle’s medium (DMEM), RPMI-1640 and bovine serum albumin (BSA) were from Sigma (St Louis, MO), and fetal bovine serum (FBS) from Thermo scientific. Rabbit anti α-SMA, anti-CD16 and anti-CD14 bound micromagnetic beads were purchased from Miltenyi Biotec (Auburn, CA), mouse anti-human E-cadherin antibody from BD Biosciences (Mississauga, ON, Canada), and anti-TGF-β_1_ monoclonal antibody (mAb) (1D11) from R&D Systems (Minneapolis, MN). Sepasol-RNA I super G (Nacalai tesque), anti-mouse antibodies against CD11b (integrin α_M_), CD49d (integrin α_4_), CD29 (integrin β_1_), CD18 (integrin β_1_), CD54 (ICAM-1), and CD106 (VCAM-1) were from BioLegend.

### Cell lines

BEAS-2B, an adenovirus 12-SV40 virus hybrid (Ad12SV40) transformed human epithelial cells, was obtained from the Riken Cell Bank (Tsukuba, Japan), and cultured in DMEM supplemented with 10% (v/v) heat-inactivated FBS, 0.03% (w/v) L-glutamine, 100 IU/ml penicillin and 100 μg/ml streptomycin. EoL-1 cells were obtained from the Riken Cell Bank, maintained in suspension culture at 37 °C and 5%CO_2_ in humidified atmosphere using RPMI-1640 medium supplemented with 10%(v/v) heat-inactivated FBS, 0.03%(w/v) L-glutamine, 100 IU/ml penicillin and 100 μg/ml streptomycin. For differentiation, EoL-1 cells were diluted to 5 × 10^5^ cells/ml and 0.5 mM sodium n-butyrate (BA) was added. EoL-1 cells were incubated with 0.5 mM BA for 5 days.

### Preparation of human eosinophils

Eosinophils from healthy human volunteers (age 30 to 45 years old with no present history of any disease) were purified by negative selection using anti-CD16 and anti-CD14 bound micromagnetic beads as previously described [19]. The purity of eosinophils was more than 97% as measured by the Randoph’s phloxine-methylene blue stain [21].

### Co-culture experiment and morphological analysis

BEAS-2B cells were cultured in 6- or 12-well plates until 60–70% cell confluence, then serum-starved for 24 h. Eosinophils were pre-treated with procaterol (provided by Otsuka Pharmacy) at 10^−9^ M for 1 h. Human eosinophils (1 × 10^6^ cells for 12-well plate, 2 × 10^6^ cells for 6-well plate) were added to the culture RPMI medium and incubated for further 24 h. After co-culture, human eosinophils were removed from adherent BEAS-2B cells by gentle pipetting. BEAS-2B cells were stained by Diff-Quick technique and photographed for analyzing morphological changes. For immunofluorescence, cells were fixed with 4% paraformaldehyde for 10 min at room temperature and stained with mouse anti-E-cadherin mAb and anti α-SMA Ab (rabbit polyclonal) followed by the secondary antibodies (donkey anti-mouse IgG conjugated with AF488 and chiken anti-rabbit IgG conjugated with AF594. Deparaffinized tissue sections were subjected to hydrated autoclaving for antigen retrieval. After washing with Tris-buffered saline, slides were exposed to mouse anti–human E-cadherin antibody (1:200) overnight at 4 °C and subsequently incubated with donkey anti-mouse IgG-Alexa Fluor 488 (1:200) for 4 h at room temperature after washing. Staining of α-SMA was done using rabbit anti–human α-SMA antibody (1:200) and then chicken anti-rabbit IgG-Alexa Fluor 594 (1:200). After washing, the sections were counterstained with 4,6-diamidino-2-phenylindole (DAPI) and mounted using a fluorescence mounting medium.

In separate experiments, human eosinophils (2 × 10^5^ cells) were prepared and treated with 10^−7^ M procaterol or 10^−5^ M forskolin (Nacalai Tesque, Kyoto, Japan) for 30 min at 37 °C. A group of eosinophils was then co-cultured with serum-starved semi-confluent BEAS-2B cells (2.5 × 10^5^ cells/well, 12-well plate) for 24 h and the cell surface expression of integrins on eosinophils was evaluated by flow cytometry. Control eosinophils were cultured alone for 24 h. Another group of eosinophils was co-cultured with BEAS-2B cells for 48 h and the cell supernatants and adherent cells (BEAS-2B cells) were collected for analysis of cytokine expression by RT-PCR and immunoassays.

### Reverse transcriptase polymerase chain reaction (RT-PCR)

After co-culture of BEAS-2B cells and eosinophils for 24 h, eosinophils were removed as described above. Total RNA was extracted from BEAS-2B cells by the guanidine isothiocyanate procedure using Trizol Reagent. RNA was reverse-transcribed using oligo-dT primers and then the DNA was amplified by PCR. The sequences of the primers are as follows: for human vimentin, forward 5′-GAGAACTTTGCCGTTGAAGC-3′ and reverse 5′-GCTTCCTGTAGGTGGTGGCAATC-3′; for human E-cadherin forward: 5′-GTATCTTCCCCGCCCTGCCAATCC-3′ and reverse 5′-CCTGGCCGATAGAATGAGACCCTG-3′; for human GAPDH, forward 5′-GTGAAGGTCGGACTCAACGGA-3′ and reverse 5′-GGTGAAGACGCCAGTGGACTG-3′. PCR was carried for 35 cycles (E-cadherin), 27 cycles (Vimentin), 25 cycles (GAPDH), denaturation at 94 °C for 30s, annealing at 65 °C for E-cadherin and GAPDH, and 59 °C for vimentin for 30s, and elongation at 72 °C for 1 min: at the end of these cycles, a further extension was carried out at 72 °C for 5 min. The PCR products were separated on a 2% agarose gel containing 0.01% ethidium bromide. The RNA concentration and purity were determined by UV absorption at 260:280 using an Ultrrospec 1100 pro UV/Vis spectrophotometer (Amersham Biosciences, NJ). The amount of mRNA was normalized against the GAPDH mRNA.

### Immunoassays

The immunoassay kit for measuring transforming growth factor (TGF)-β1 (R&D, McKinley Place, MN) and granulocyte-macrophage colony-stimulating factor (GM-CSF) were purchased from BD Biosciences Pharmingen (San Jose, CA); and each parameter was measured following the manufacturer’s instructions.

### Statistical analysis

All data were expressed as the mean ± standard error of the mean (S.E.M.). The statistical difference between two variables was calculated by the Mann–Whitney *U* test, and that between three or more variables by one-way analysis of variance with Dunnett’s test. We used the software package GraphPad Prism 6 (GraphPad Software, San Diego, CA) for all statistical analyses. *P* < 0.05 was considered as statistically significant.

## Results

### Procaterol inhibits EMT induced by human EoL-1 cells

**Fig. 1:**
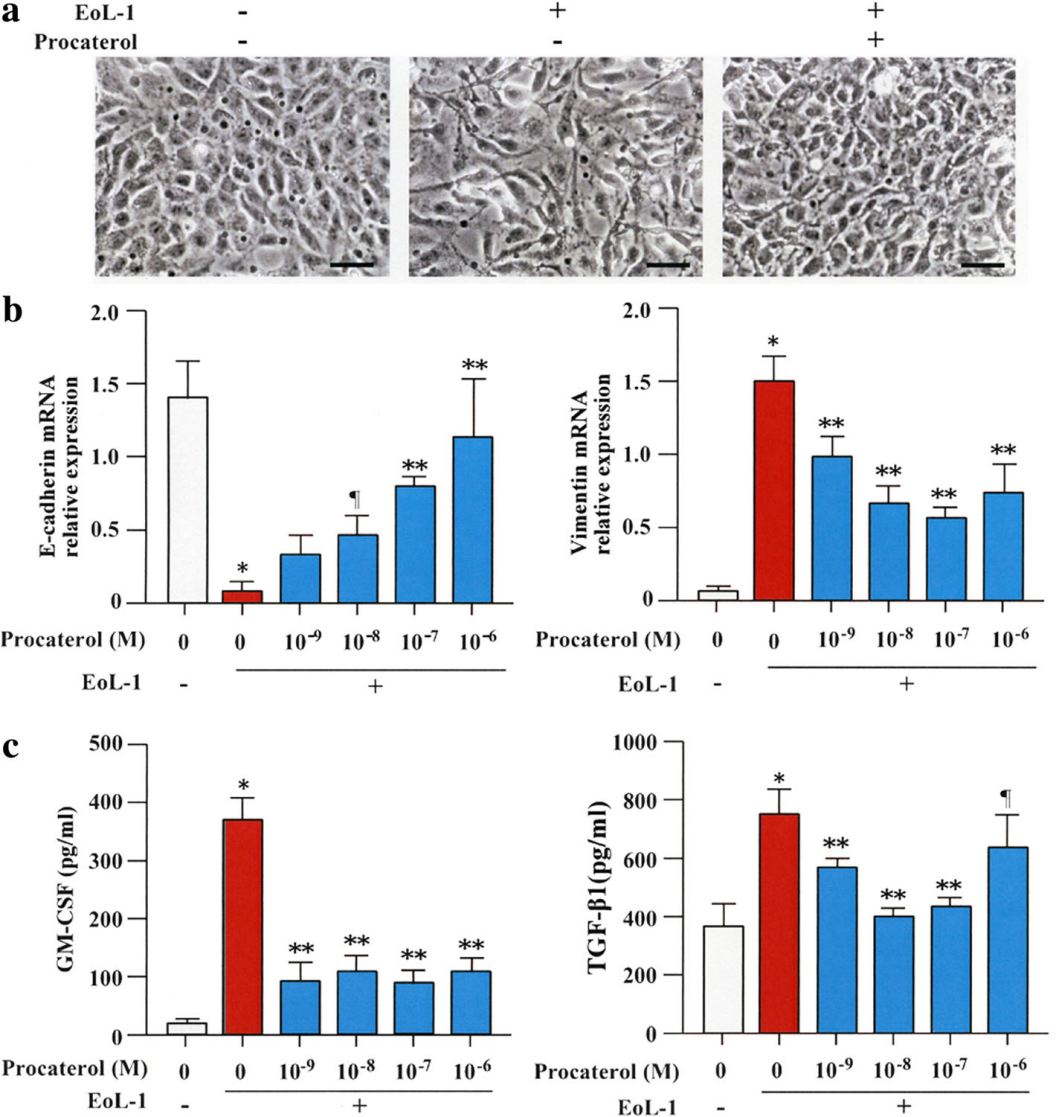
EMT induced by EoL-1 is inhibited by procaterol. **a** Control and BEAS-2B cells co-cultured with EoL-1 in the presence or absence of procaterol (original magnifications, ×400). **b** Gene expression of E-cadherin and vimentin in BEAS-2B cells co-cultured with EoL-1 in the presence (10^−9^ M ~ 10^−6^ M) or absence of procaterol as evaluated by RT-PCR. **c** Granulocyte-macrophage colony-stimulating factor (GM-CSF) and transforming growth factor (TGF-β1) levels in the supernatant after co-culture in the presence (10^−9^ M ~ 10^−6^ M) or absence of procaterol. Bars indicate mean ± SEM. Scale bars indicate 100 μm. The data are the representative of a single experiment performed in triplicates. Two independent experiments were performed. *p < 0.0001 vs procaterol 0 group; **p < 0.001 and ¶*p* < 0.05 vs EoL-1(+)/procaterol (−) group. Statistics by analysis of variance with Dunnett’s test

BEAS-2B cells were co-cultured with EoL-1 in the presence or absence of procaterol. BEAS-2B cells cultured in medium alone conserved the typical epithelial cobblestone pattern, but BEAS-2B cells co-cultured with EoL-1 presented fibroblast-like morphology consistent with EMT (Fig. 1a). Procaterol inhibited these morphological changes. RT-PCR analysis showed that procaterol significantly inhibited the decrease in the expression of the epithelial marker E-cadherin and the increase in the expression of the mesenchymal marker vimentin in BEAS-2B cells co-cultured with EoL-1 in a concentration-dependent manner (Fig. 1b). Pre-treatment with procaterol significantly and dose-dependently inhibited the increase of TGF-β1 and GM-CSF in the culture supernatant sampled during co-culture of BEAS-2B cells with human EoL-1 cells (Fig. 1c).

Subsequent investigations were performed using procaterol at concentration of 10^−7^ M because the optimal effective concentration of procaterol in human is between 10^−8^ M ~ 10^−7^ M.

### Procaterol inhibits EMT induced by primary human eosinophils

**Fig. 2:**
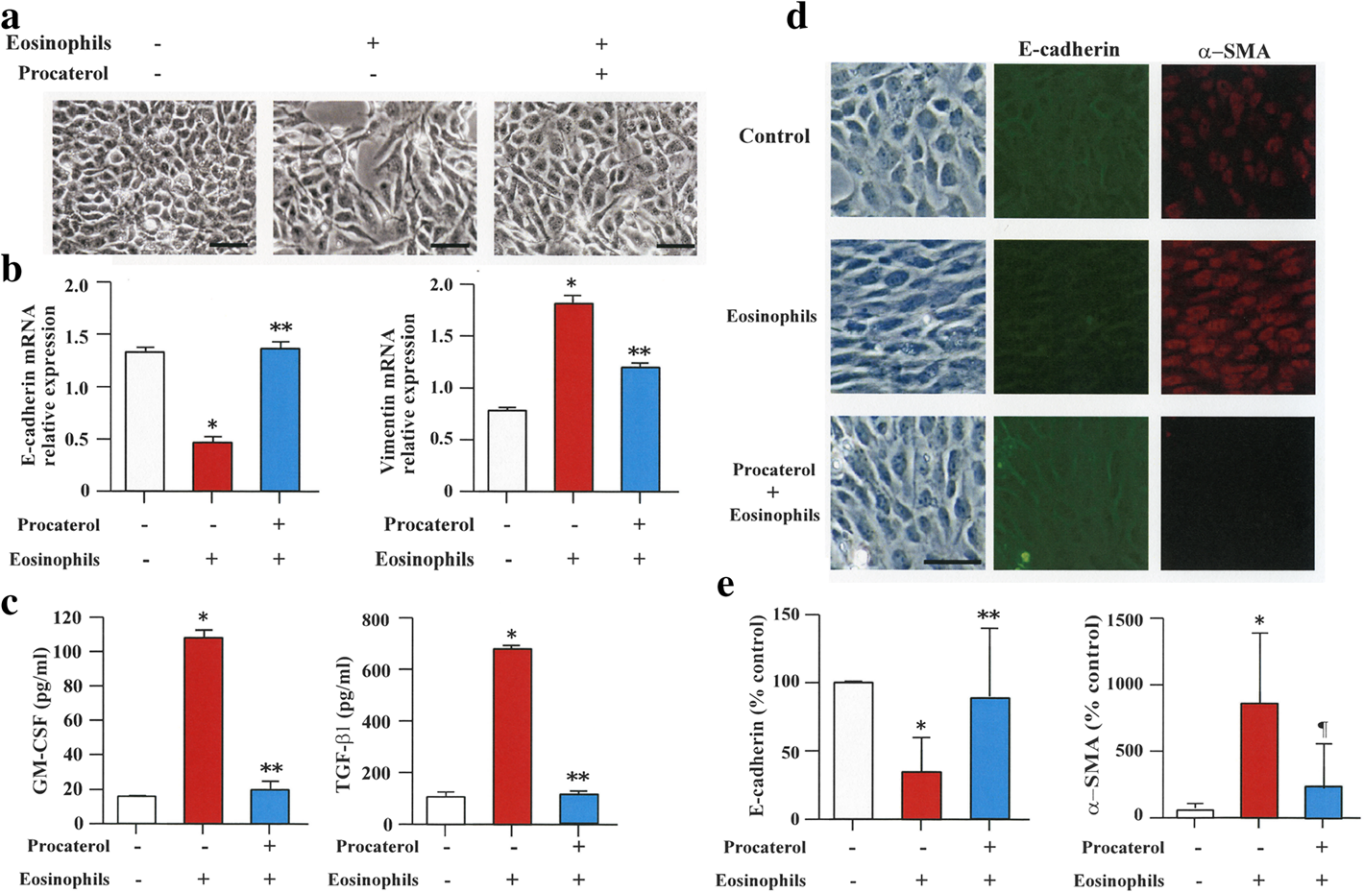
EMT induced by primary human eosinophils is inhibited by procaterol. **a** Control and BEAS-2B cells co-cultured with primary human eosinophils in the presence or absence of procaterol (original magnifications, ×400). **b** Gene expression of E-cadherin and vimentin as assessed by RT-PCR. **c** Granulocyte-macrophage colony-stimulating factor (GM-CSF) and transforming growth factor (TGF-β1) levels in the supernatant. **d** Representative immunofluorescence staining of E-cadherin (green) and α-SMA (red) in BEAS-2B cell with saline or eosinophils or eosinophils pre-treated with procaterol. **e** Quantification by densitometry. Bars indicate mean ± SEM. Scale bars indicate 100 μm. The data are the representative of a single experiment performed in triplicates. Two independent experiments were performed. *p < 0.001 vs procaterol (−) group; **p < 0.001 and ¶*p* < 0.05 vs eosinophils (+)/procaterol (−) group. Statistics by analysis of variance with Dunnett’s test

BEAS-2B cells were co-cultured with primary human eosinophils in the presence or absence of procaterol. BEAS-2B cells co-cultured with human eosinophils exhibited fibroblast-like morphology consistent with EMT, but this was inhibited when human eosinophils were pre-treated with procaterol. BEAS-2B cells cultured in medium alone conserved the typical epithelial cobblestone pattern, but BEAS-2B cells co-cultured with human eosinophils showed spindle forms; culture in the presence of procaterol inhibited these morphological changes (Fig. 2a). RT-PCR analysis showed that procaterol significantly inhibited the decrease in the expression of E-cadherin and the increased expression of vimentin in BEAS-2B cells co-cultured with human eosinophils (Fig. 2b). Pre-treatment with procaterol significantly inhibited the increase of TGF-β1 and GM-CSF in the supernatant obtained during co-culture of BEAS-2B cells with human eosinophils (Fig. 2c).

Immunofluorescence staining of E-cadherin (green) and α-SMA (red) in BEAS-2B cell was also performed. Procaterol significantly inhibited the decrease in the expression of E-cadherin and the increase in the expression of α-SMA in BEAS-2B cells co-cultured with human eosinophils (Fig. 2d, e).

### Butoxamine, a specific β_2_-adrenergic antagonist, inhibits the effect of procaterol

**Fig. 3:**
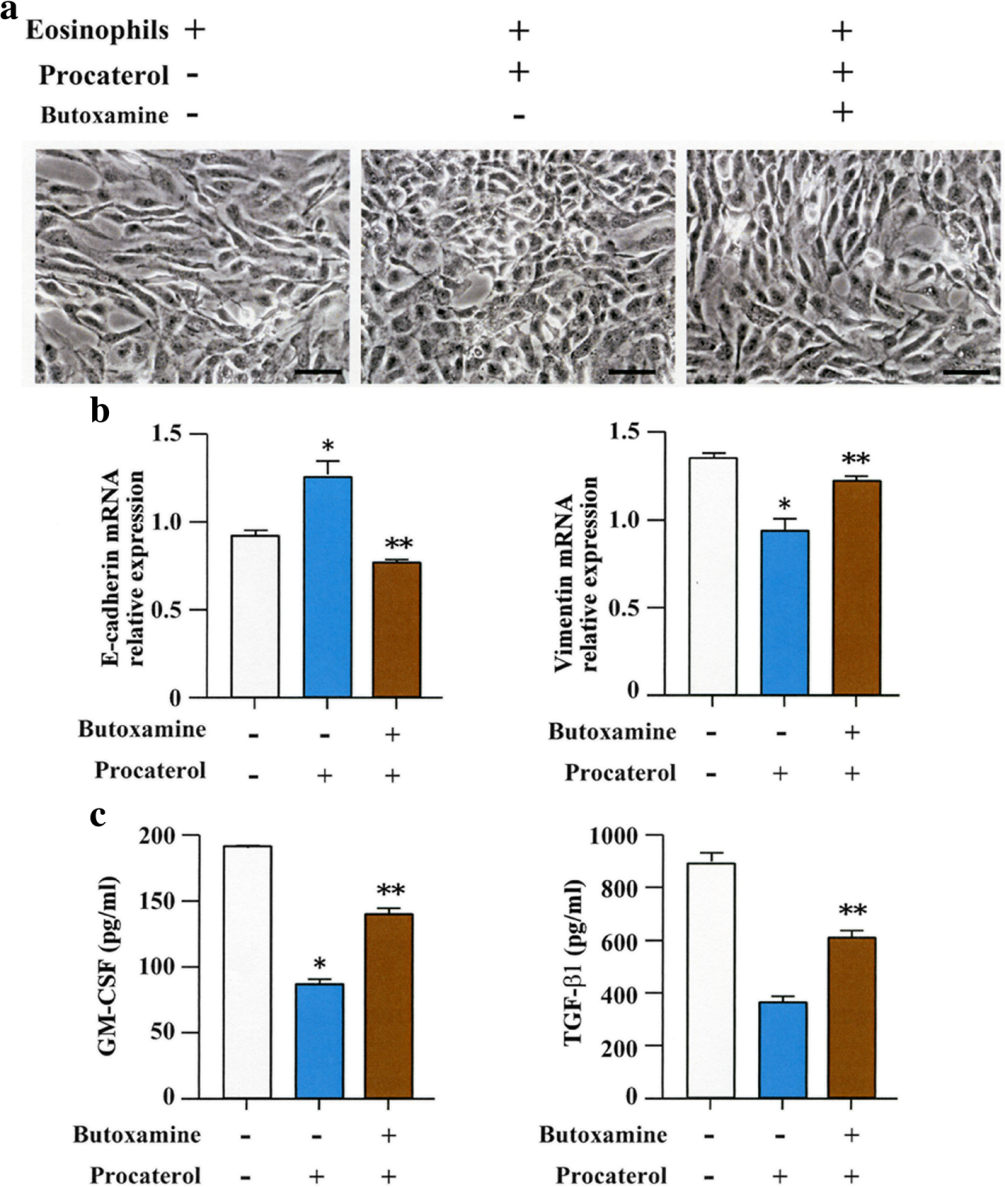
A specific β_2_ adrenergic receptor inhibitor blocks the effect of procaterol. **a** Control and BEAS-2B cells co-cultured with human eosinophils in the presence or absence of procaterol and butoxamine (original magnifications, ×400). **b** Gene expression of E-cadherin and vimentin as evaluated by RT-PCR. **c** Granulocyte-macrophage colony-stimulating factor (GM-CSF) and transforming growth factor (TGF-β1) levels in the supernatant. Bars indicate mean ± SEM. Scale bars indicate 100 μm. The data are the representative of a single experiment performed in triplicates. Three independent experiments were performed. *p < 0.005 vs butoxamine (−)/procaterol (−) group; **p < 0.005 vs butoxamine (−)/procaterol (+) group. Statistics by analysis of variance with Dunnett’s test

BEAS-2B cells pretreated with butoxamine before adding procaterol, and co-cultured with human eosinophils showed fibroblast-like morphology (Fig. 3a). RT-PCR analysis showed that butoxamine significantly inhibited the expression of E-cadherin and vimentin in BEAS-2B cells co-cultured with human eosinophils (Fig. 3b). Pre-treatment with butoxamine significantly blocked changes induced by procaterol on secretion of TGF-β_1_ and GM-CSF in the cell supernatant during co-culture of BEAS-2B cells with human eosinophils (Fig. 3c).

### Procaterol inhibits the expression of adhesion molecules

**Fig. 4:**
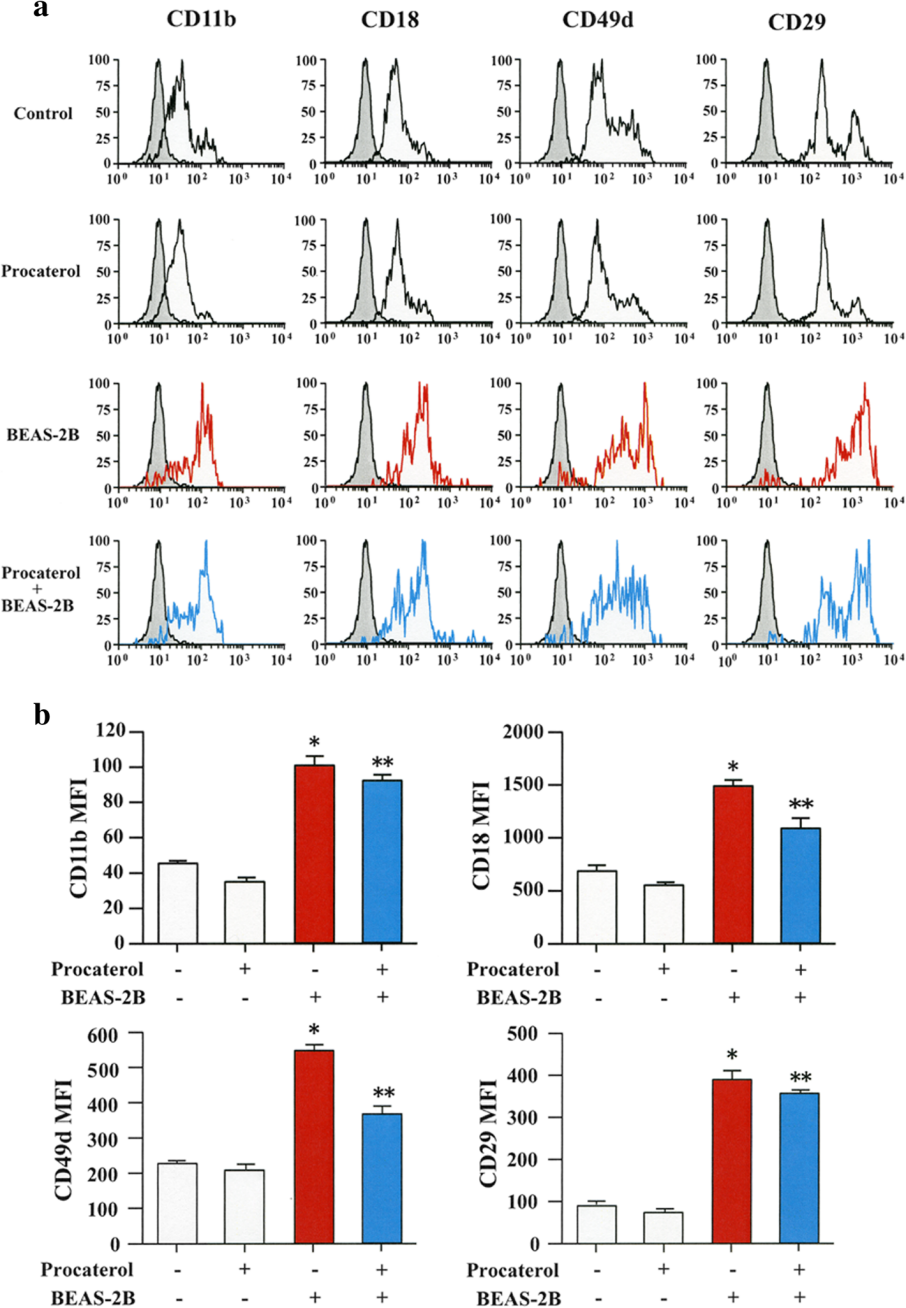
Procaterol inhibits the expression of adhesion molecules from BEAS-2B cells co-cultured with EoL-1 cells. **a** The expression of ICAM-1 and VCAM-1 on BEAS-2B cells co-cultured with EoL-1 cells in the presence or absence of procaterol as analyzed by flow-cytometry. **b** Quantification by MFI. Bars indicate mean ± SEM. The data are the representa tive of a single experiment performed in triplicates. Two independent experiments were performed.*p < 0.001 vs EoL-1 (−)/procaterol (−) group; **p < 0.05 vs EoL-1 (+)/procaterol (−) group. Statistics by analysis of variance with Dunnett’s test

We have already reported the need of eosinophil contact to induce EMT of bronchial epithelial cells, thus we analyzed the expression of adhesion molecules on eosinophils by flow cytometry. The expression of the adhesion molecules ICAM-1 and VCAM-1 on BEAS-2B cells co-cultured with EoL-1 cells were enhanced in the absence of procaterol but it was inhibited when EoL-1 cells were pretreated with procaterol before co-culturing with BEAS-2B cells (Fig. 4a, b).

**Fig. 5:**
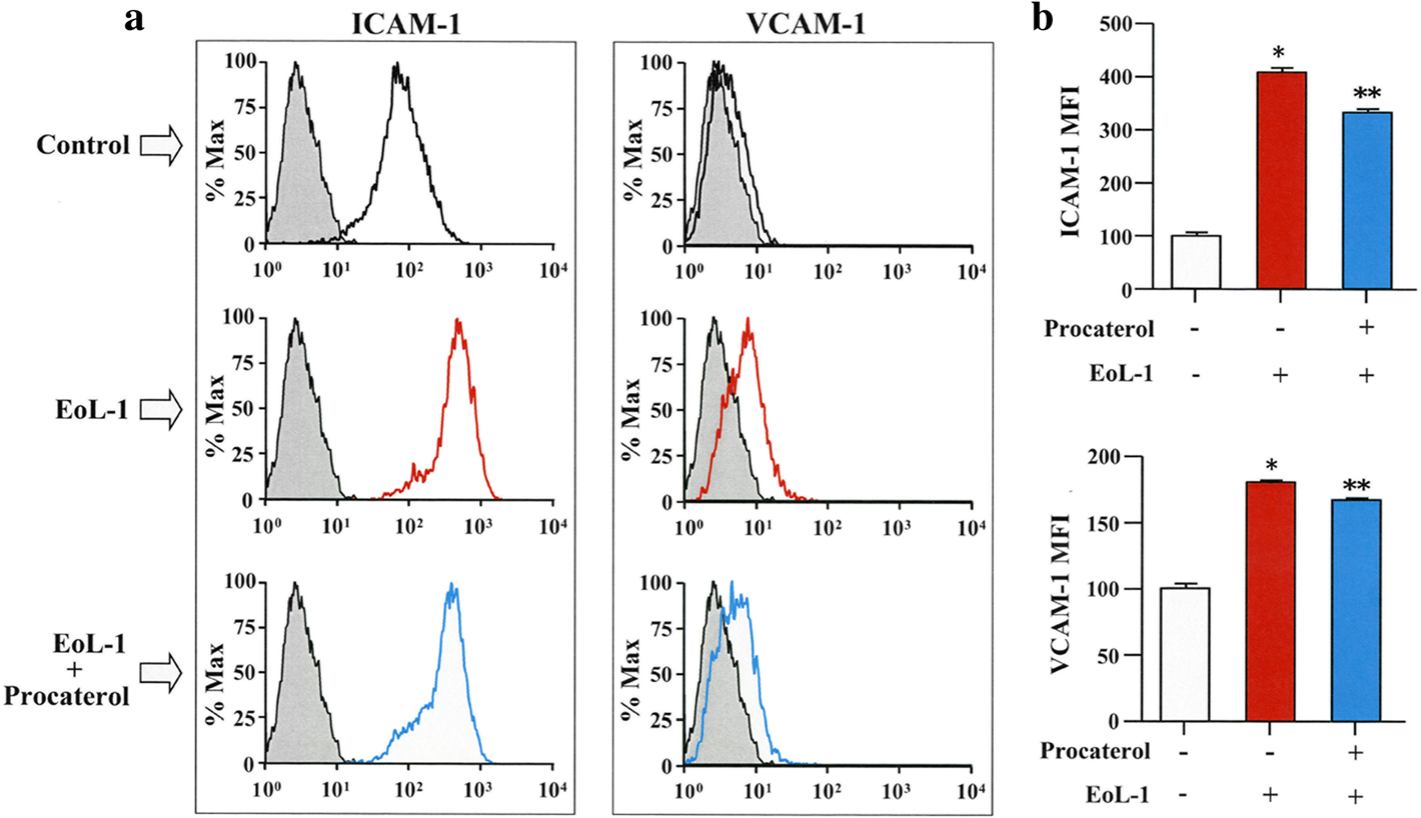
Procaterol inhibits the expression of adhesion molecules on human eosinophils co-cultured with BEAS-2B cells. **a** The expression of αM (CD11b), β2 (CD18), α4 (CD49d), β1 (CD29) integrin subunits on eosinophils as analyzed by flow cytometry after co-culture with BEAS-2B cells and eosinophils in the presence or absence of procaterol. **b** Quantification by MFI. Bars indicate mean ± SEM. The data are the representative of a single experiment performed in triplicates. Two independent experiments were performed. *p < 0.05 vs BEAS-2B (−)/procaterol (−) group; **p < 0.05 vs BEAS-2B (+)/procaterol (−) group. Statistics by analysis of variance with Dunnett’s test

The expressions of α4(CD49d), β1(CD29), αM(CD11b) and β2(CD18) integrin subunits were also evaluated during co-culture in the presence or absence of procaterol. The expression of CD49d, CD29, CD11b and CD18 were strongly enhanced when BEAS-2B cells were co-cultured with eosinophils pretreated without procaterol, but they were significantly inhibited when eosinophils were pretreated with procaterol (Fig. 5a, b).

### Suppression of EMT by antibodies against integrin and/or anti-adhesion molecules

**Fig. 6:**
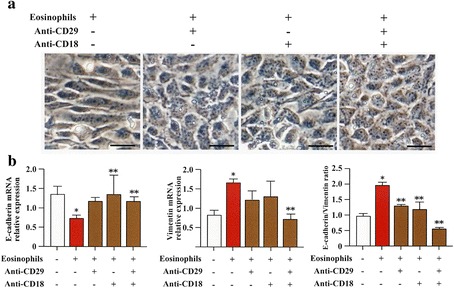
EMT induced by eosinophils is suppressed by anti-integrin antibodies. **a** BEAS-2B cells co-cultured with human eosinophils in the presence of anti-integrin antibodies (anti-CD18 Ab and/or anti-CD29 Ab). **b** Gene expression of E-cadherin and vimentin in BEAS-2B cells co-cultured with human eosinophils in the presence or absence of anti-integrin antibodies (anti-CD18 Ab and/or anti-CD29 Ab). Bars indicate mean ± SEM. Scale bars indicate 100 μm. The data are the representative of a single experiment performed in triplicates. Two independent experiments were performed. *p < 0.01 vs eosinophils (−)/anti-CD29 (−)/anti-CD18 group; **p < 0.05 vs eosinophils (+)/anti-CD29 (−)/anti-CD18 group. Statistics by analysis of variance with Dunnett’s test

The role of adhesion molecules in EMT during co-culture was evaluated. The characteristic morphological changes of EMT in BEAS-2B cells co-cultured with eosinophils were abolished in the presence of anti-integrin antibodies (anti-CD18 Ab and/or anti-CD29 Ab) (Fig. 6a). Anti-integrin antibodies also significantly inhibited the decreased expression of E-cadherin, and the increased expression of vimentin (Fig. 6b).

**Fig. 7:**
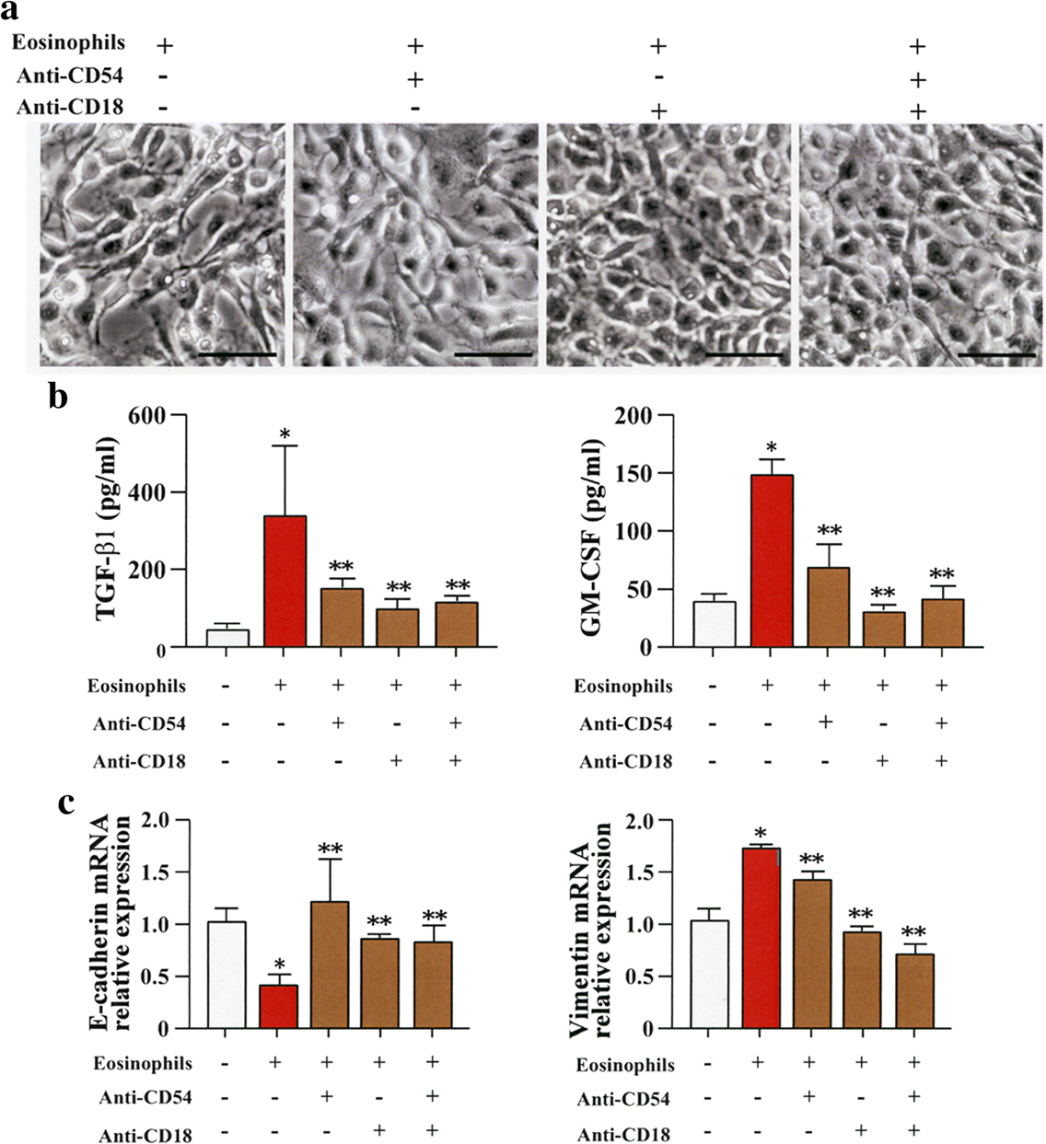
EMT is suppressed by anti-integrin antibody and/or anti-adhesion molecule antibody. **a** BEAS-2B cells co-cultured with human eosinophils in the presence of anti-integrin antibodies and/or anti-ICAM-1 antibodies (anti-CD18 Ab and/or anti-CD54 Ab). **b** Granulocyte-macrophage colony-stimulating factor (GM-CSF) and transforming growth factor (TGF-β1) levels in the supernatant. Bars indicate mean ± SEM. **c** Scale bars indicate 100 μm. The data are the representative of a single experiment performed in triplicates. Two independent experiments were performed. Gene expression of E-cadherin, and vimentin in BEAS-2B cells co-cultured with eosinophils. *p < 0.01 vs eosinophils (−)/anti-CD54 (−)/anti-CD18 group; **p < 0.05 vs eosinophils (+)/anti-CD54 (−)/anti-CD18 group. Statistics by analysis of variance with Dunnett’s test

EMT of BEAS-2B cells was inhibited in the presence of anti-ICAM-1 antibody (anti-CD54 Ab) (Fig. 7a). Anti-ICAM-1 antibody significantly inhibited the inhibitory effect of procaterol on the expression of TGF-β_1_ and GM-CSF during co-culture of BEAS-2B cells with human eosinophils (Fig. 7b). The decreased expression of E-cadherin, and the increased expression of vimentin (Fig. 7c) were also significantly inhibited by anti-ICAM-1 antibody (Fig. 7c).

### Forskolin exerts similar effects of procaterol

**Fig. 8:**
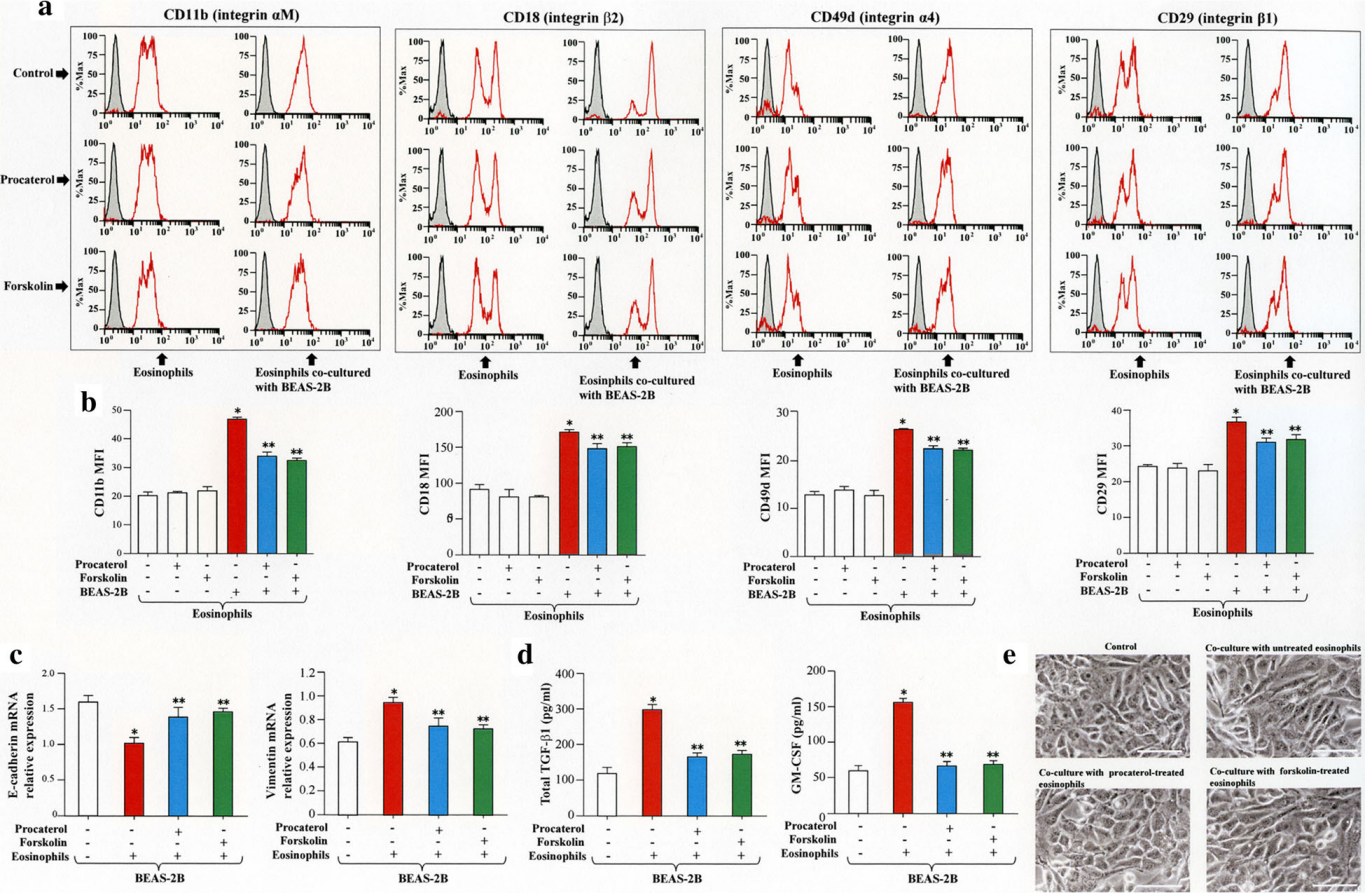
Forskolin and procaterol have similar effects. **a**, **b** Human eosinophils were pre-treated with procaterol or forskolin for 30 min and then co-cultured with serum-starved BEAS-2B for 24 h before analyzing integrin expression by flow cytometry. **c** BEAS-2B cells were collected to evaluate the mRNA expression of E-cadherin and vimentin. **d** Co-culture supernatants were collected to evaluate the levels of transforming growth factor (TGF-β1) and granulocyte-macrophage colony-stimulating factor (GM-CSF). **e** EMT of BEAS-2B cells were evaluated in each treatment group. Bars indicate mean ± SEM. Scale bars indicate 100 μm. The data are the representative of a single experiment performed in triplicates. *p < 0.05 vs control groups; **p < 0.05 vs eosinophils co-cultured with BEAS-2B in the absence of both procaterol and forskolin. Statistics by analysis of variance with Dunnett’s test

To demonstrate that increased intracellular levels of cyclic adenosine monophosphate (cAMP) is critical for the inhibitory activity of procaterol, we evaluated whether similar effects can be observed with forskolin, a well-recognized activator of adenylyl cyclase. As expected the surface expression of integrins (CD11b, CD18, CD49d, CD29) on eosinophils co-cultured with BEAS-2B cells was significantly inhibited by forskolin compared to control cells (Fig. 8a, b). In addition, the mRNA expression of E-cadherin was significantly increased while that of vimentin was significantly decreased in BEAS-2B cells co-cultured with eosinophils treated with forskolin compared to control cells (Fig. 8c). The concentrations of TGF-β1 and GM-CSF were also significantly suppressed in the co-culture supernatant in the presence of forskolin compared to control (Fig. 8d). EMT was also inhibited in epithelial cells co-cultured in the presence of eosinophils pre-treated with procaterol or forskolin (Fig. 8e).

## Discussion

The results of this study provides the first evidence that procaterol, a selective and full β_2_-agonist, suppresses EMT of bronchial epithelial cells induced by eosinophils.

### Adhesion molecules and airway remodeling

EMT of airway epithelial cells plays an important role in airway remodeling associated chronic bronchial asthma [22–26]. Mesenchymal cells during EMT migrate to the subepithelial connective tissue where they produce extracellular matrix proteins and contribute to airway wall fibrosis [27]. We previously reported that direct contact of eosinophils with the BEAS-2B cells increases the expression of TGF-β_1_ and induces EMT [20]. Hansel et al. reported that adhesion molecules on eosinophils play crucial roles in bronchial asthma [28]. We found that neither increase in the level of supernatant TGF-β_1_ nor induction of EMT occurs when the cells are cultured using a trans-well system suggesting the need of cell contact. Adhesion molecules play a critical role in cell-to-cell interaction [20]. Here, we showed that co-culture of epithelial cells and eosinophils up-regulates the expression of integrins on eosinophils and ICAM-1 and VCAM-1 on epithelial cells, and that inhibition of integrin-mediated cell-contact inhibits EMT of epithelial cells. Integrin-mediated signaling in eosinophils appears to induce the production of TGF-β1 leading to EMT of epithelial cells. In the present study, we found that procaterol inhibits the expression of adhesion molecules from eosinophils and that EMT is suppressed in the presence of anti-adhesion molecule antibodies during co-culture of bronchial epithelial cells with primary eosinophils. Inhibition of the expression of adhesion molecules appears to be associated with increased intracellular cyclic AMP activation [6]. In support of this, we found that the effect of forskolin, a cAMP-promoting agent, is similar to that of procaterol. A previous study has shown that suppression of RhoA activation by increased intracellular levels of cAMP inhibits integrin-dependent adhesion of leukocytes [29]. Therefore, it is conceivable that elevation of intracellular levels of cAMP is the mechanism by which procaterol decreases activation of eosinophils leading to downregulated expression of integrin molecules and TGF-β1 in eosinophils making them less capable of inducing EMT. All together, these observations suggest that procaterol suppresses eosinophil-induced EMT by blocking the expression of adhesion molecules on eosinophils. It is worth noting that, in addition to eosinophils, other cells including macrophages and neutrophils are also capable of inducing EMT [30, 31].

### Bronchoconstriction and TGF-β_1_ expression

β_2_ adrenergic agonists are the first line drug for relief of acute asthma symptoms and a long-term controller in combination with inhaled corticosteroids [2]. They are the key bronchodilators used in the reversal of acute bronchospasm of bronchial asthma and for the treatment of COPD [1, 2]. These agonists may also have important anti-inflammatory effects on eosinophils in airway chronic diseases [7]. Grainge et al. showed that bronchoconstriction without additional inflammation induced airway remodeling in patients with asthma [32]. They found that bronchoconstriction induced by either allergen or methacholine increases TGF-β_1_ production from the airway epithelium. This previous study also provided evidence that repeated bronchoconstriction increases the thickness of the sub-epithelial collagen layer, which is an early indicator of airway collagen deposition and epithelial mesenchymal signaling [32]. In the present study, TGF-β_1_ secretion was suppressed by procaterol. Thus prevention of airway contraction by using β_2_ agonists may lead to amelioration of airway remodeling.

### Study limitations

The purity of eosinophils was not 100%, and thus EMT could have been caused by hematopoietic cells rather than eosinophils. However, in a previous study we demonstrated that eosinophils isolated using the same method, but not contaminating cells, promote EMT in the model used here [19]. The fact that EMT induced by a eosinophil cell line (Eol-1) was inhibited by procaterol also supports the role of human eosinophils in our present model of EMT. The lack of an in vivo study is another limitation; but we already reported that eosinophils play an important role in airway remodeling in vivo and that procaterol at a clinical dose reduces eosinophil inflammation [19]. Therefore, it is likely that suppression of eosinophils-induced EMT by procaterol is a relevant mechanism even in vivo.

## Conclusions

In summary, this study showed that procaterol, β_2_ adrenergic agonists, suppresses eosinophils-induced EMT of airway epithelial cells, and this finding may explain the mechanism by which β_2_ adrenergic agonists ameliorate airway remodeling in chronic obstructive pulmonary diseases including bronchial asthma.

## Abbreviations

EMT: Epithelial-to-mesenchymal transition
GM-CSF: Granulocyte-macrophage colony-stimulating factor
ICAM-1: Intercellular Adhesion Molecule 1
mAb: Monoclonal antibody
TGF-β1: Transforming growth factor
VCAM-1: Vascular cell adhesion molecule 1
